# Electroosmotic Flow Induced Lift Forces on Polymer Chains in Nanochannels

**DOI:** 10.1021/acspolymersau.1c00058

**Published:** 2022-03-08

**Authors:** Lisbeth Perez Ocampo, Lisa B. Weiss, Marie Jardat, Christos N. Likos, Vincent Dahirel

**Affiliations:** † Sorbonne Université, CNRS, Physico-chimie des électrolytes et nano-systèmes interfaciaux, PHENIX, F-75005 Paris, France; ‡ Faculty of Physics, University of Vienna, Boltzmanngasse 5, A-1090 Vienna, Austria

**Keywords:** nanofluidics, electroosmotic flow, Poiseuille flow, polymers, hydrodynamics

## Abstract

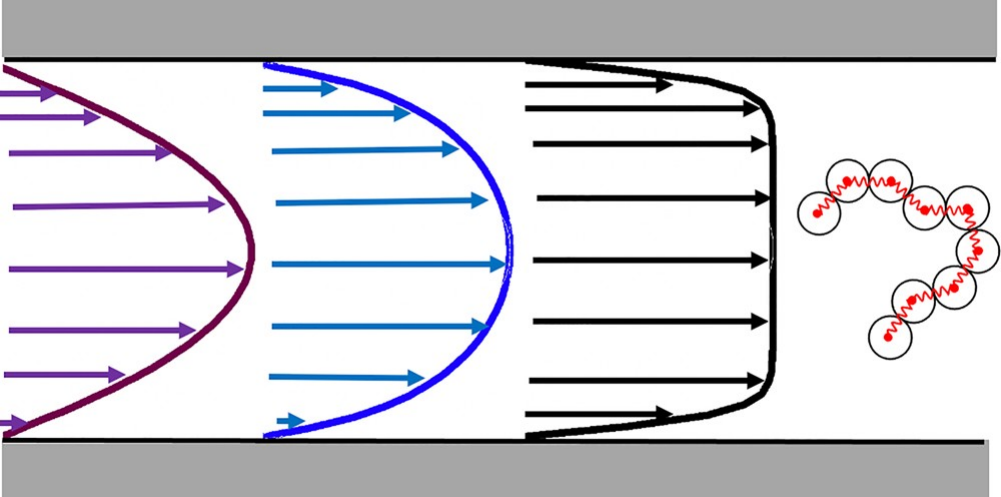

A major objective of research in nanofluidics is to achieve better selectivity in manipulating the fluxes of nano-objects and in particular of biopolymers. Numerical simulations allow one to better understand the physical mechanisms at play in such situations. We performed hybrid mesoscale simulations to investigate the properties of polymers under flows in slit pores at the nanoscale. We use multiparticle collision dynamics, an algorithm that includes hydrodynamics and thermal fluctuations, to investigate the properties of fully flexible and stiff polymers under several types of flow, showing that Poiseuille flows and electroosmotic flows can lead to quantitatively and qualitatively different behaviors of the chain. In particular, a counterintuitive phenomenon occurs in the presence of an electroosmotic flow: When the monomers are attracted by the solid surfaces through van der Waals forces, shear-induced forces lead to a stronger repulsion of the polymers from these surfaces. Such focusing of the chain in the middle of the channel increases its flowing velocity, a phenomenon that may be exploited to separate different types of polymers.

## 1 Introduction

Polymers are ubiquitous in soft materials: they are used in paints and in food products as shear thinning and gelling agents respectively; they act as foam stabilizers in skin care products; they carry the genetic information (DNA and RNA) in all living cells; and they are also found in soils, as a product of the biodegradation of organic matter where they can affect the transport of pollutants.

Polymer physics describes the generic (universal) properties of polymers.^1,2^ The shape of polymer molecules is constantly fluctuating, and these fluctuations can be characterized through the distribution of coarse-grained variables, such as the polymer instantaneous gyration radius or its asphericity.^3,4^ At equilibrium, these variables are related to enthalpic and entropic forces, both of which can be influenced by the presence of interfaces. Under confinement, the constraints on polymer degrees of freedom usually give rise to effective entropic repulsions from the interfaces. Indeed, when the center of the polymer gets close to an interface, the 3D polymer chain needs to extend on the 2D surface, and it loses entropy. The resulting entropic forces can nevertheless be overcome by enthalpic ones, such as electrostatic or van der Waals (vdW) interactions with the interface atoms.

The aforementioned polymer properties may be strongly affected in nonequilibrium situations, where external forces apply on individual monomers or on the surrounding solvent.^5,6^ Purely nonequilibrium hydrodynamic forces^7−9^ are then superimposed to the typical equilibrium, entropic, and enthalpic forces that act on the monomers. In micro- or nanofluidic devices, polymer solutions can be purely sheared or the whole solution can be transported. In this case, the hydrodynamic constraint on the polymer is a key determinant of polymer transport through the channel. We are not interested here in Couette flow, for which the shear rate does not depend on the polymer position. Such a situation has been extensively studied, and in particular the influence of the flow on the polymer internal structure has been characterized though scaling laws^3,5,6,10,11^ and finite size effects;^12−15^ these studies include extensions to more complex systems, such as star^16^ and ring polymers.^17,18^ Here, we focus on cases for which the hydrodynamic constraints depend on the distance to the channel walls.

The above considerations are particularly important at the nanoscale, where functionalities benefiting from predominance of surfaces (e.g., nanofluidic transistors and diodes) can be developed.^19^ A number of recent discoveries has highlighted the enormous potential of nanofluidics and membranes made of novel nanomaterials, such as carbon (CNT) or boron nitride (BNT) nanotubes, as well as graphene or related materials. BNTs, for instance, allow to harvest the energy contained in salinity gradients with an exceptional efficiency,^20^ suggesting that they could be used as highly competitive membranes to harvest the chemical energy contained in the difference of salinity between seawater and river water, the so-called osmotic power or blue energy. Water transport through nanoscale pores is of fundamental importance to many natural systems, such as biological ion channels and zeolites, and it affects numerous technologies, including molecular level drug delivery, energy efficient nanofiltration, and chemical detection. As water invariably contains salt, the dissociated salt ions are bound to play a crucial role in transport properties both of the pure solvent and of possible dissolved entities, such as polymers. Driving the solvent flow via pressure gradients leads to the usual Poiseuille flow whereas imposing an electric field that acts on the dissociated ions results into electroosmotic flow.

Under Poiseuille flow, polymer tumbling and deformation give rise to effective *lift* forces that vanish when the flow driving force is switched off.^21,22^ These effective forces push the polymer in the direction perpendicular to the wall, toward the center of the channel. This results in a flow dependent positioning of the polymer in the channel, often referred to as *hydrodynamic focusing*.^7−9^ The driving mechanism is related to the shear rate in the fluid embedding the polymer. Under a typical Poiseuille flow, for a polymer whose gyration radius *R*_g_ is only a fraction of the distance between walls *L*_*z*_ (e.g., *L*_*z*_ = 4*R*_g_), monomers have a stationary hydrodynamic velocity that depends on their position within the chain. This shall cause an extension of the chain and its overall tumbling.^11,15,23−25^ This may effectively repel the polymer from the wall. The key hydrodynamic number that characterizes this out of equilibrium dynamics is the Weissenberg number *Wi*, expressed as the ratio between a polymer relaxation time scale^5,13^ (e.g., the relaxation time scale for internal degrees of freedom or the equilibrium diffusion time scale) and a time scale related to the hydrodynamic constraints, and in particular to the local shear rate γ̇ of the fluid^14,25^ that drives the polymer motion (stretching, tumbling, and focusing). The relaxation time scale can be given, e.g., by the Rouse theory (Rouse time) if hydrodynamic interactions between monomers are negligible or by the Zimm theory (Zimm time) if hydrodynamics is not screened.^26^ If the shear induced motion of the polymer is faster than its relaxation time scale, then the structural quantities characterizing the polymer shape remain different from those at equilibrium while it tumbles, resulting into a stronger hydrodynamic focusing.^8^

Such phenomena are difficult to predict analytically. Indeed, a combination of several elements shall be accurately described: the structure of the polymer at equilibrium,^3,27^ the impact of shear and hydrodynamic interactions between monomers on the folding of the polymer, the impact of collisions with the wall when the polymer tumbles, and so on. This difficulty increases if one wants to study the interplay between lift forces and other kinds of interactions with the wall, as well as the influence of intrachain constraints (e.g., correlations between bond vectors).^28,29^ In order to understand the leading physical mechanisms at play, the resort to atomistic simulations is not necessary. The model should include hydrodynamics and thermal fluctuations for a self-avoiding chain within the typical universality classes of polymer physics. Mesoscopic simulations are therefore particularly adapted techniques to unravel the key physical phenomena underlying the properties of polymers under flow.

Our work is motivated by the need to understand the ways in which the interplay between various solvent flow profiles, polymer characteristics, and monomer–wall interactions can lead to different scenarios regarding the focusing of the polymer away from the channel walls, affecting thereby the transportation speed of the chain along the microfluidic device. Controlling the spatial dependence of the shear opens the possibility to steer the balance between nonequilibrium forces and conservative ones (e.g., arising from van der Waals attractions to the wall, or intrapolymer constraints). For instance, if we impose a strong shear rate close to the interface simultaneously with an attractive van der Waals force, their coupling could qualitatively change the behavior of the polymer under confinement. When several types of mechanisms can compete with each other, simulations are precious tools to switch on the different mechanisms independently. In the present study, we aim at performing a systematic comparison of the effect on the polymer behavior under confinement and under flow of a van der Waals attraction by the walls, and of the polymer stiffness, using multiparticle collision dynamics (MPCD) simulations.

The paper is organized as follows: in section 2, we present the physical setup of the microfluidic channel, our approach to simulate electroosmotic flow, the polymer models invoked, as well as the interactions of the monomers with the channel walls. In section 3, we summarize our hydrodynamic model of choice, namely, the MPCD algorithm, whereas our results are discussed in section 4. Finally, in section 5, we summarize and draw our conclusions.

## 2 Flowing Nanosystems under Investigation

### 2.1 Model Flows in Slit Pores

The freedom and flexibility offered by a flow generated through a pressure gradient (Poiseuille flow) are rather limited. For a carrier fluid of a fixed density and viscosity, only the strength of the external force and the boundary conditions on the wall can be tuned. If the maximum velocity (i.e., the value of the external field) is fixed, the velocity profile within the channel has a fixed parabolic shape (and a fixed linear shear rate profile), which can only be shifted if the boundary conditions vary from stick to slip conditions. While lift forces have been characterized for polymers under Poiseuille flow,^7−9^ there is not much diversity of shear effects that can be obtained, as long as we stay in the low Reynolds number regime. Moreover, in many nanodevices, the pressure gradient that is necessary to make the fluid flow within the nanoporosity is not achievable.

Alternative flow driving forces can be used, such as capillary forces, or electrostatic ones. In biological systems, electrokinetic flows are thought to play a key role in transporting macromolecules. In the current study, we investigate hydrodynamic focusing under an electroosmotic flow. Electroosmotic (EO) flow occurs in charged porous systems in the presence of an external electric field, which acts on the net mobile electric charge due to the electric double layer. Compared to the Poiseuille flow, the electroosmotic flow gives additional degrees of freedom to manipulate the velocity profile, through the control of the salt concentration or of the ionic valency, which modify the electric double layer. In the case of EO flows, the *z*-dependence of the shear rate can be tuned independently from the maximum velocity. In other words, the shear rate can be more or less *concentrated* close to the wall when the salt concentration varies. In the absence of added salt, i.e., when the only mobile charges are the counterions of the charged solid surfaces, the flow profile is similar to the Poiseuille one. In the presence of an added salt, it evolves toward a plug flow when the ionic concentration increases. This implies that new physical phenomena can arise that are not seen under a Poiseuille flow, and that might have interesting consequences in the design of micro- and nano- fluidic systems to manipulate polymers.

### 2.2 Electroosmotic Flow

**Figure 1:**
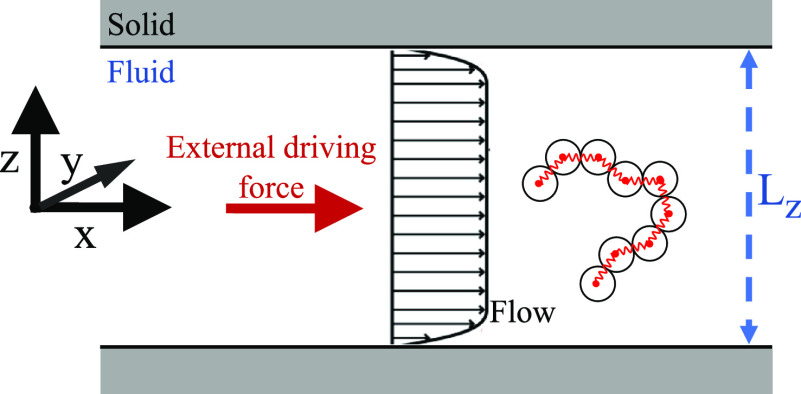
Geometry of the simulated systems. As an illustration, an electroosmotic flow is represented, pertaining to the case with added salt.

We simulate flowing fluids between two parallel hard walls, with no slip boundary conditions. This channel is studied at the nanometric scale. Using realistic parameter values for our coarse-grained model, we find that our hard walls are separated by distances *L*_*z*_ = 2.5 nm and *L*_*z*_ = 5.0 nm for the two different setups we considered. Periodic boundary conditions are applied in the two directions *x*,*y* parallel to the walls. In all cases, the fluid is transported in the *x* direction. The overall geometry is sketched in Figure 1.

In the following, we define as Poiseuille flow a mass flow driven by a pressure gradient within the system. In a periodically repeated system, it is impossible to set up a pressure gradient, as the average pressure must remain constant in the flow direction *x*. In numerical simulations, the Poiseuille flow is therefore generated by applying a constant force to all fluid particles in the system. We define as electroosmotic flow (EO flow) a transport flow that is generated by an external electric field applied in a channel with an electrostatic double layer. In order to generate an EO flow, an electrostatic force in the *x* direction shall be applied to all charged particles of the system. Explicit ion MPCD simulations are computationally expensive, even if they can be used in order to simulate EO flows^30^ and to study the influence of small ions on the properties of suspensions of charged nanoparticles.^31^ In what follows, we propose to induce an EO flow by applying an external force on the fluid without explicitly describing surface charges and ions. Indeed, in the EO flow, the momentum creation due to the electrostatic force is locally transmitted to the fluid particles surrounding the charged solutes. The electrostatic force is effectively applied to the whole fluid as a function of the local charge density, and this force is exactly compensated by viscous forces at the stationary state. If the charge density ρ_el_(*z*) is known, one may create an EO flow by applying a local force distribution ρ_el_(*z*)*E* to the fluid.

To investigate the influence of the type of flow on the behavior of the polymer, we make systematic comparisons between three types of stationary flows: (i) the reference Poiseuille flow, (ii) a typical electroosmotic flow with a pluglike shape, which occurs when there is added salt in the flowing fluid (the added salt concentration is always *c*_salt_ = 2 mol L^–1^), and (iii) an electroosmotic flow in the case without added salt. In order to make the comparison meaningful, we keep the spatial average Γ̇ of the shear rate γ̇(*z*) (=∂*v*/∂*z*) identical in all cases:
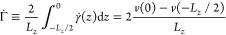
1with *v*(*z*) being the velocity profile of the fluid between walls located at *z* = ±*L*_*z*_/2. In the case with no slip boundary conditions (*v*(−*L*_*z*_/2) = *v*(*L*_*z*_/2) = 0), this situation corresponds to a fixed maximum velocity of the fluid *v*(0) = *v*_max_. In all cases, we compare two maximum velocities, while staying in the low Reynolds number regime.

The flow is in all cases generated using a body force within the MPCD fluid. For the Poiseuille flow, the body force is constant in all the fluid phase, similar to a gravitational force. For the electroosmotic flow, the force depends on the distance to the wall: It is a force distribution ρ_el_(*z*) *E* that is related to a theoretical electric charge distribution ρ_el_(*z*). We used the nonlinearized Poisson–Boltzmann theory to compute the charge distribution ρ_el_(*z*) between two infinite parallel walls with a surface charge Σ = 0.5 *e* nm^–2^ (with *e* the elementary charge) in both cases with and without added salt. The computation of the charge distribution requires the definition of an electrostatic length scale, the Bjerrum length *l*_B_. This length corresponds to the distance where the Coulombic interaction energy between two monovalent ions equals the thermal energy *k*_B_*T*, where *k*_B_ is Boltzmann’s constant and *T* the temperature, depending thus on the temperature and on the solvent dielectric constant. We set the Bjerrum length at its value for water at room temperature, *l*_B_ = 0. 718 nm. As we previously showed, an electroosmotic flow generated by MPCD simulations with explicit ions leads to the same shear distribution, as long as the charge distribution ρ_el_(*z*) is valid.^30^

### 2.3 Polymer Model and Polymer–Wall Interactions

We consider different types of model polymers, moving within a slit nanoporous medium. They differ through their stiffness and through their interaction with the walls of the pore. Each simulated system contains a single polymer chain, made of *N* = 40 monomers. The monomers are coupled to the MPCD fluid using the collisional coupling rule, as described in the following section.

The first polymer model is a freely jointed chain of monomers, with vibrating monomer–monomer bonds. The interaction potential between monomers is characterized by a characteristic length σ, which defines the size of the monomer. This potential has two parts.^32^ A Weeks–Chandler–Andersen (WCA) repulsive potential *U*_WCA_(*r*) is employed to model good solvent conditions, and it acts between all monomers:
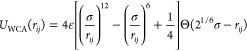
2where ε is the interaction strength, chosen here to be equal to the thermal energy, ε = *k*_B_*T*, *r*_*ij*_ is the distance between two monomers *i* and *j*, and Θ is the Heaviside function. Moreover, connectivity between successive monomers is provided by a FENE potential *U*_FENE_(*r*):^33^
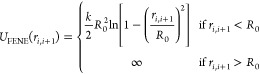
3with the standard Kremer–Grest parameters for the bond constant *k* = 30ε/σ^2^ and the maximum bond extension *R*_0_ = 1.5 σ to prevent nonphysical bond-crossing.^34^ This potential has the form of a simple harmonic potential for small *r*_*i*,*i*+1_, but it limits the spring extension to *R*_0_.

In addition to this flexible-chain model, we study a simple model of stiff polymers, by adding a bending potential (semiflexible chains). The potential energy of a semiflexible chain described by a set of bond angles θ_*i*_ in a given conformation is given by
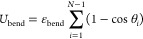
4where ε_bend_ is the bending energy. In this model, the persistence length of the polymer *L*_p_ can be deduced from the parameters of the bending potential as *L*_p_ = 2σ/⟨θ^2^⟩, the denominator expressing the strength of the fluctuations of the bond angle around its expectation value ⟨θ⟩ = 0. Our model of stiff polymer has a persistence length *L*_p_ ≅ 10σ.

We study two classes of interactions between monomers and walls. In the first class, monomers interact with the wall through purely repulsive potentials. In this case, the only role of the interaction is to exclude the polymer from the solid phase. It is effectively represented through the Stochastic Reflection Rule (SRR) described in the next section. In the second class, in addition to the effective repulsive interaction, monomers interact with the wall through van der Waals (vdW) attractions. The charges of the wall and of the mobile ions are only implicitly described through their influence on the EO flow, but their screening effect should be included in the vdW potential. This can be approximated by using a Yukawa term exp(−κ*r*) acting on the induced dipole vdW term of the form *V*_vdW_(*r*) = −*w*σ^6^/*r*^6^, with a typical strength *w* having dimensions of energy. Here, κ is the inverse Debye screening length, defined by the equation:
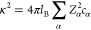
5where *Z*_α_ and *c*_α_ are respectively the valency and the concentration of the ionic solute of type α. Moreover, the energy parameter *w* of the vdW potential includes the dielectric constant ε_r_ of the solvent. The attraction shall be integrated over the whole solid volume. Nevertheless, we assume a localization of induced dipoles at the wall–fluid boundary (walls at the position *z*_*w*_ = −*L*_*z*_/2 and *z*_*w*_ = *L*_*z*_/2), avoiding the integration of the potential *V*_vdW_(*r*) over *z*, leading to a laterally integrated van der Waals interaction *U*_vdW_(*z*) expressed as the sum of the contributions from the two walls:
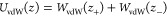
6with *z*_±_ = *L*_*z*_/2 ± *z* being the distance to the wall at *z*_*w*_ = ∓*L*_*z*_/2 and
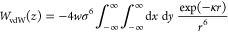
7This leads to the following explicit expression for the van der Waals attraction to a single wall that implicitly includes the influence of the ionic solution:
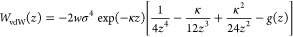
8with
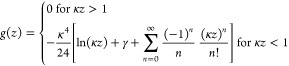
9In what follows, we set *w* = *k*_B_*T*.

## 3 Computational Methods

### 3.1 Multiple Particle Collision Dynamics

Multiparticle collision dynamics (MPCD) is a mesoscopic method that has already widely been used for polymers.^35−37^ It has proven to be adapted to simulate polymer dynamics in the Zimm regime, where hydrodynamic interactions are predominant.^38^ A highly simplified solvent is simulated, which is simple enough to ensure computational efficiency. It enables to generate thermal noise in the system, although an additional thermostat is needed in nonequilibrium situations. The algorithm locally conserves momentum while creating fluctuations, and it is thus equivalent to a Navier–Stokes solver with thermal noise. The MPCD solvent transmits momentum within the system through streaming and effective collisions, and therefore the simulation can mimic various hydrodynamic regimes.^35,39^ When a solute is included in the fluid, MPCD is much more appropriate than Navier–Stokes solvers, since the explicit nature of the solvent makes it easier to couple to the moving solute. From a structural perspective, at equilibrium, MPCD allows to obtain the exact structure of a given model of a polymer system, as would do a Monte Carlo simulation. Also, MPCD allows one to study hydrodynamic problems with complex boundaries, and it is thus an attractive method for interacting polymers under flow. First studies could unravel the behavior of concentrated polymers between walls,^35^ whereas recently is has been shown how polymers of different topologies, e.g., ring or linear polymers, can be separated under Poiseuille flow.^37^

Two steps are involved in MPCD. In a *streaming step*, positions and velocities of each fluid particle *i* are propagated by integrating Newton’s equations of motion. A second step, the *collision step*, enables local momentum exchanges between the fluid particles. The simulation box is partitioned into cubic collision cells of edge length *a*_0_. In each cell, the velocities of fluid particles relative to the velocity of the center of mass of the cell are rotated by an angle α around a randomly oriented axis. The angle α is a fixed parameter. A random shift of the collision grid is performed at each collision step to ensure Galilean invariance.^35,40^ It is convenient to use the fluid particle mass *m*_f_ as the mass unit, the size of the collision cells *a*_0_ as the length unit, and *k*_B_*T* as the energy unit. The time unit is then
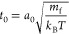
10

This fluid can be coupled to solute particles in various ways. Within the *collisional coupling* scheme, solute particles interact with each other through a classical force field and participate to the collision step with solvent particles. Details about this simulation scheme can be found in several reviews.^35,41^ The clear advantage of this coupling method is that it is very efficient from a computational point of view. One drawback is that, as some of us showed in a recent article,^42^ the hydrodynamic radius *a*_hyd_ of solute particles is almost constant at the scale of the MPCD collision cell size *a*_0_, of the order of 0.3*a*_0_. In the case of polymers in the Zimm regime, this is not an issue as the effect of the size of the monomers on the hydrodynamic behavior is not relevant.^26^ The temperature of solvent particles and monomers was controlled by employing a cell-level Maxwellian thermostat. This thermostat ensures constant temperature and solvent particle densities over the complete channel volume in addition to the correct Maxwell–Boltzmann distribution for the relative solvent particle velocities.^43^

To reproduce no-slip boundary conditions at the surface of the walls, we use the SRR algorithm. It was first proposed by Inoue et al.^44^ and later refined by Padding et al.^45^ (we use here the latter). Briefly, within this scheme, when a solvent particle enters the solid phase, the time and position of the impact is computed and the solvent particle is restored to this impact point and is given a random velocity obtained through a half-plane Maxwell–Boltzmann distribution. Within this methodology, it is not necessary to divide the streaming step into smaller MD steps for the solvent particles.^46^

### 3.2 Parameters of the MPCD Simulations

The parameters related to the solvent are chosen to reproduce hydrodynamic interactions typical of a liquid (as opposed to a gas). Following Ripoll et al.,^41^ we chose the rotation angle α = 130°, the average solvent number density ρ = 5*a*_0_^–3^, and the collision time step δ*t*_c_ = 0.1*t*_0_. For this choice of parameters, the kinematic viscosity of the fluid is ν = 0.81 *a*_0_^2^*t*_0_^–1^, so that for the dynamic viscosity we obtain η = 4.05*m*_f_*a*_0_^–1^*t*_0_^–1^. The solutes, which are here coupled through the MPCD fluid during the collision steps, are dynamically characterized through their mass. We take here a monomer mass M = 5*m*_f_, which is the average mass of solvent fluid particles within a collision cell. To avoid divergence of the energy of the simulation box, the MD step δ*t*_MD_ used to integrate the equation of motion for solutes is smaller than the time step δ*t*_c_ between two collisions. Here, δ*t*_MD_ was empirically chosen based on the stability of the total energy of the system, from 0.02δ*t*_c_ to 0.01δ*t*_c_ depending on the system. For every set of parameters, with or without flow, at least four distinct independent trajectories were done, allowing us to compute error bars.

The typical size of a monomer σ, as defined in the interaction potentials, is equal to the size of the collision cells *a*_0_, as it has been done in other polymer studies.^35,37^ The value of the inverse Debye length κ depends on the system: For no added salt, κ = 0.23*a*_0_^–1^, and for *c*_salt_ = 2 mol L^–1^, κ = 0.47*a*_0_^–1^. The distance between the walls is either 17 or 34 times the size of the monomer bead (*L*_*z*_ = 17*a*_0_ or *L*_*z*_ = 34*a*_0_). The other dimensions of the simulation box are *L*_*x*_ = 60*a*_0_, *L*_*y*_ = 45*a*_0_. To create the EO flows, different values of the force distribution ρ(*z*)*eE* applied on the fluid were obtained using different external electric field intensities. To obtain the smallest maximum velocity, *v*_max,1_ = 0.411*a*_0_*t*_0_^–1^ for *L*_*z*_ = 34*a*_0_, we took *eE* = 40*m*_f_*a*_0_*t*_0_^–2^ (without salt) and *eE* = 76 *m*_f_*a*_0_*t*_0_^–2^ (with added salt). To obtain the largest maximum velocity, *v*_max,2_ = 1.644*a*_0_*t*_0_^–1^ for *L*_*z*_ = 34*a*_0_, we took *eE* = 160 *m*_f_*a*_0_*t*_0_^–2^ (without salt) and *eE* = 307 *m*_f_*a*_0_*t*_0_^–2^ (with added salt). To create the Poiseuille flow, the applied external body force distribution was *f* = 0.0461 *m*_f_*a*_0_^–2^*t*_0_^–2^ in the case of *L*_*z*_ = 34*a*_0_, *v*_max,2_ = 1.644*a*_0_*t*_0_^–1^. Values of the applied force in other cases shall be deduced from the relation *v*_max_ = (*fL*_*z*_^2^)/(8η).

### 3.3 Computation of Structural Parameters

The polymer structure is described through the computation of the following quantities. First, the linear distribution *c*(*z*) of monomers along the *z* axis is used to characterize the hydrodynamic focusing within the channel. This is simply the probability density of finding the center of a monomer at a given distance from the middle of the channel. Second, the internal structure of the polymer is characterized through its averaged gyration radius *R*_g_, and its asphericity. Indeed, a standard way of quantifying the typical size of a single polymer chain in a given configuration is the standard deviation of its position distribution or the instantaneous radius of gyration *R̂*_g_ defined via:^2^
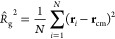
11with **r**_cm_ being the position of the center of mass:
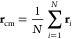
12*N* being the number of monomers of the chain, and **r**_*i*_ being the position of the *i*th monomer.

It is common to statistically characterize the average behavior of a polymer of *N* monomers by means of the mean radius of gyration,
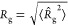
13where the average ⟨·⟩ is performed over the ensemble of conformations for a given polymer. Nevertheless, for polymers under flow, the average gyration radius per se does not help us to quantify the effect of the flow on the polymer shape. The influence of shear is better seen through the computation of the asphericity.^4^ This quantity can be deduced from the gyration tensor *Ĝ* of the polymer, whose αβ-component reads:
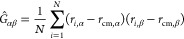
14where α,β = *x*,*y*,*z* denote Cartesian components. Since the gyration tensor is a symmetric matrix, a Cartesian coordinate system can be found in which the tensor is diagonal, where the axes are chosen such that the diagonal elements, the eigenvalues λ, are ordered: λ _1_ ≤ λ _2_ ≤ λ_3_. These diagonal elements are the principal moments of the gyration tensor and they can be combined to give several parameters. The dimensionless asphericity parameter *b* is defined as
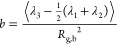
15with the denominator in eq 15 above being equal to the square of the bulk radius of gyration. Evidently, the asphericity vanishes for a perfect sphere and the deviation of a shape from sphericity is quantified by the magnitude of *b*.

## 4 Results

In order to define a reference length scale, the bulk value of the radius of gyration *R*_g,b_ for the flexible polymers was computed: *R*_g,b_ = 4.17*a*_0_. Note that we did not find a significant change in the value of the gyration radius under confinement in the absence of flow. This value of *R*_g,b_ can be compared to the channel width, either 17*a*_0_ or 34*a*_0_, which is thus of the order of a few *R*_g,b_. Moreover, the polymer is larger than the range of electrostatic interactions in the case under EO flow with added salt, as the Debye length corresponds to about 2*a*_0_ in this case. On the other hand, the gyration radius is of the order of the Debye length in the case under EO flow without salt.

### 4.1 Flow profile

**Figure 2:**
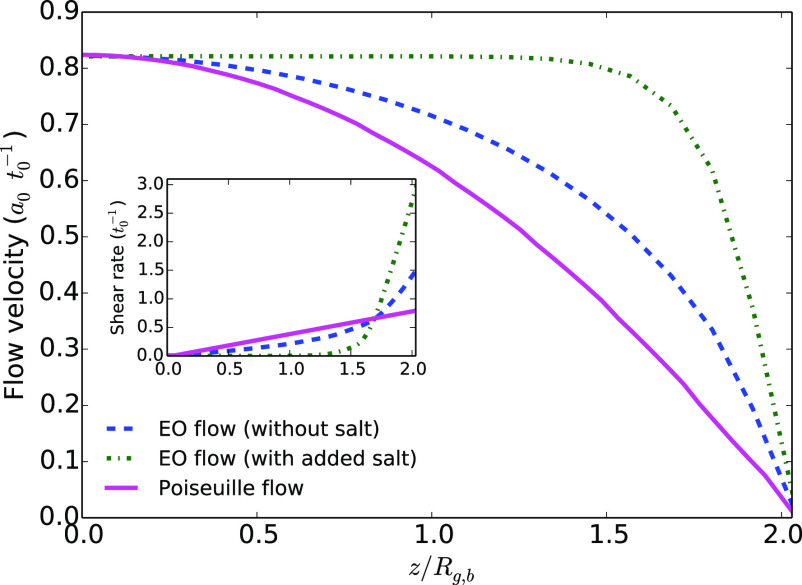
*x*-Component of the average fluid velocity, as a function of the *z* coordinate perpendicular to the walls divided by the average gyration radius of the bulk polymer *R*_g,b_. The force that creates the flow is applied in the *x*-direction. The value of the maximum velocity is here *v*_max,2_ = 0.822*a*_0_*t*_0_^–1^ for *L*_*z*_ = 17 *a*_0_. Inset: Average shear rate in the direction of the flow.

We compared the effect of three types of flows, electroosmotic (EO) flow with or without added salt and Poiseuille flow, finding that the fluid velocity profile is very weakly affected by the presence of the polymer. Two series of simulations were done, which differ on the maximum velocity of the imposed flow. For each maximum velocity, the average shear rate Γ̇, eq 1, is the same for all flows. We show in Figure 2 the velocity profiles obtained for each type of flow for the largest value of the maximum velocity. The corresponding shear rates are displayed in the inset of Figure 2. As expected, the shear rate at the wall-liquid interface is much stronger under an EO flow with added salt than under the Poiseuille flow. Halfway between the interface and the middle of the channel, the shear rate is more intense under the Poiseuille flow and it is almost zero under the EO flow with added salt. The case of the EO flow without added salt is intermediate. By passing from the Poiseuille flow to the EO flow with salt, one thus concentrates the hydrodynamic constraints at the interface.

### 4.2 Simulation of Flexible Polymers between Hard Walls

**Figure 3:**
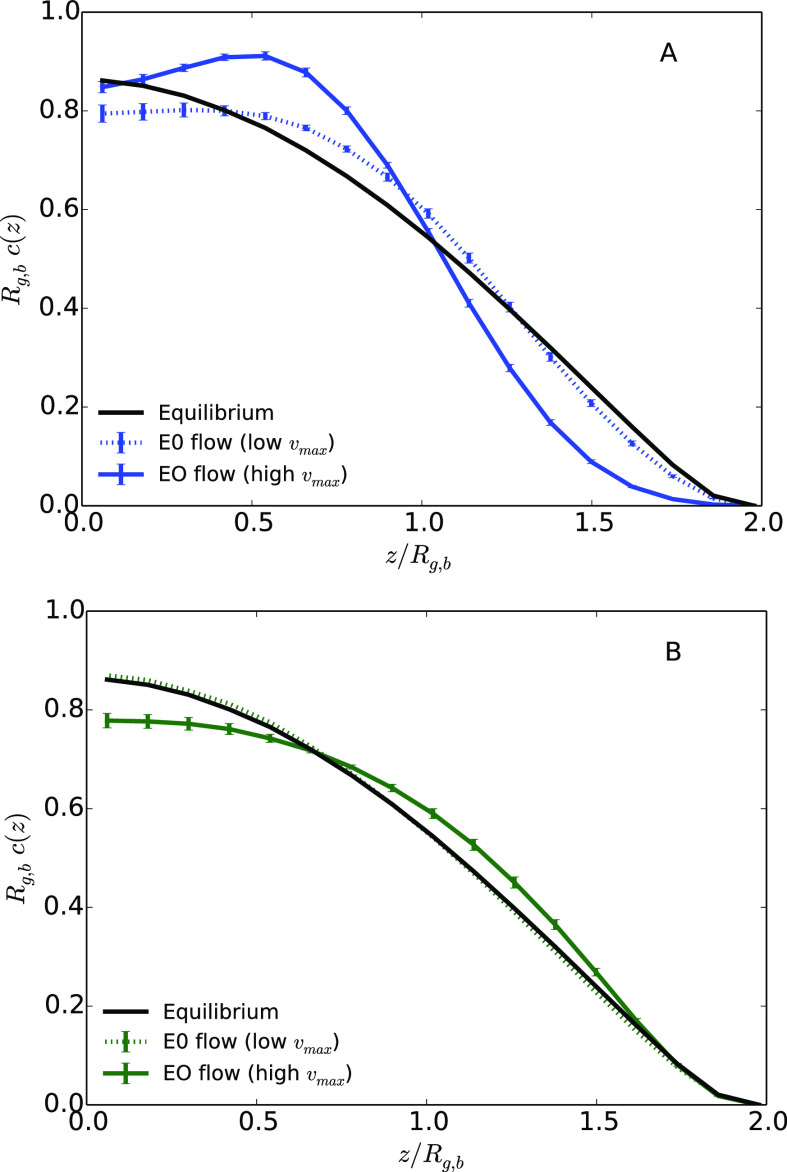
Average monomer density as a function of the distance to the center of the channel *z* divided by the average bulk gyration radius *R*_g,b_ for a distance *L*_*z*_ = 4.1*R*_g,b_ between walls. (A) Systems under an EO flow without salt; (B) systems under an EO flow with added salt.

**Figure 4:**
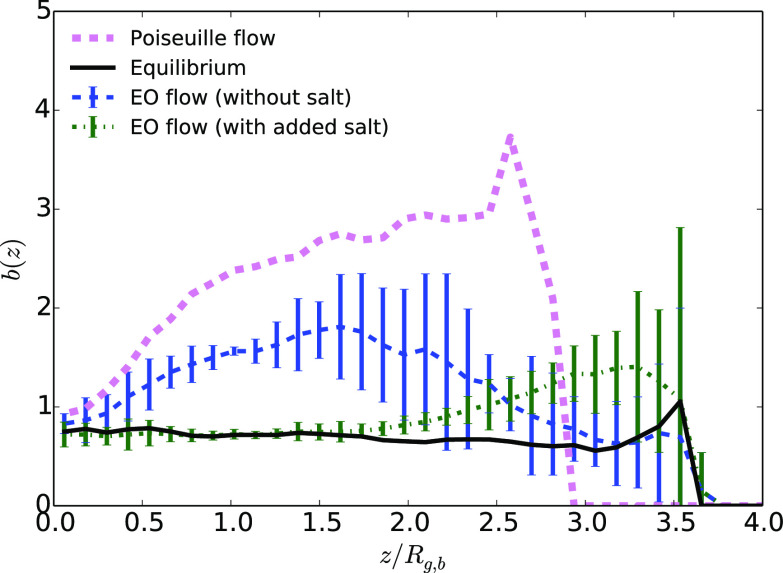
Polymer asphericity as a function of the distance to the center of the channel *z* divided by the average gyration radius in bulk, *R*_g,b_, for a distance *L*_*z*_ = 8.2*R*_g,b_ between walls at equilibrium or under flow (cases with the highest maximum velocity). The error bars on the results obtained under a Poiseuille flow are not shown, as they are high for *z*/*R*_g,b_ > 2.

In Figure 3, the average density of monomers as a function of the distance to the center of the channel is presented both at equilibrium, and under an EO flow for the two values of the maximum velocity *v*_max_, 0.411*a*_0_*t*_0_^–1^ and 0.822*a*_0_*t*_0_^–1^. Figure 3A shows the case of a polymer under an EO flow without added salt. Without added salt, the ionic charge, and thus the body force acting on the fluid, is more homogeneously distributed inside the channel than in the case with added salt. The monomer distribution clearly changes under an EO flow, and depends on the magnitude of the velocity of the fluid. The hydrodynamic focusing is clearly visible in the case of the highest maximum velocity, with a peak of the monomer distribution for a distance to the center *z* = 0.5*R*_g,b_. This behavior is qualitatively similar to what is already described in the literature under a Poiseuille flow, with a clear increase of the monomer density in the middle of the channel.^7,8^ When *z* is smaller than 0.5*R*_g,b_, the global shear on the polymer decreases since the polymer crosses the midplane (*z* = 0), and distant monomers in symmetric positions relative to this plane experience the same flow velocity. As shown in Figure 4, the polymer shape, quantified through its asphericity, is comparable to the bulk value when the polymer is at the center of the channel, explaining why hydrodynamic focusing is not maximal at the center of the channel.

**Figure 5:**
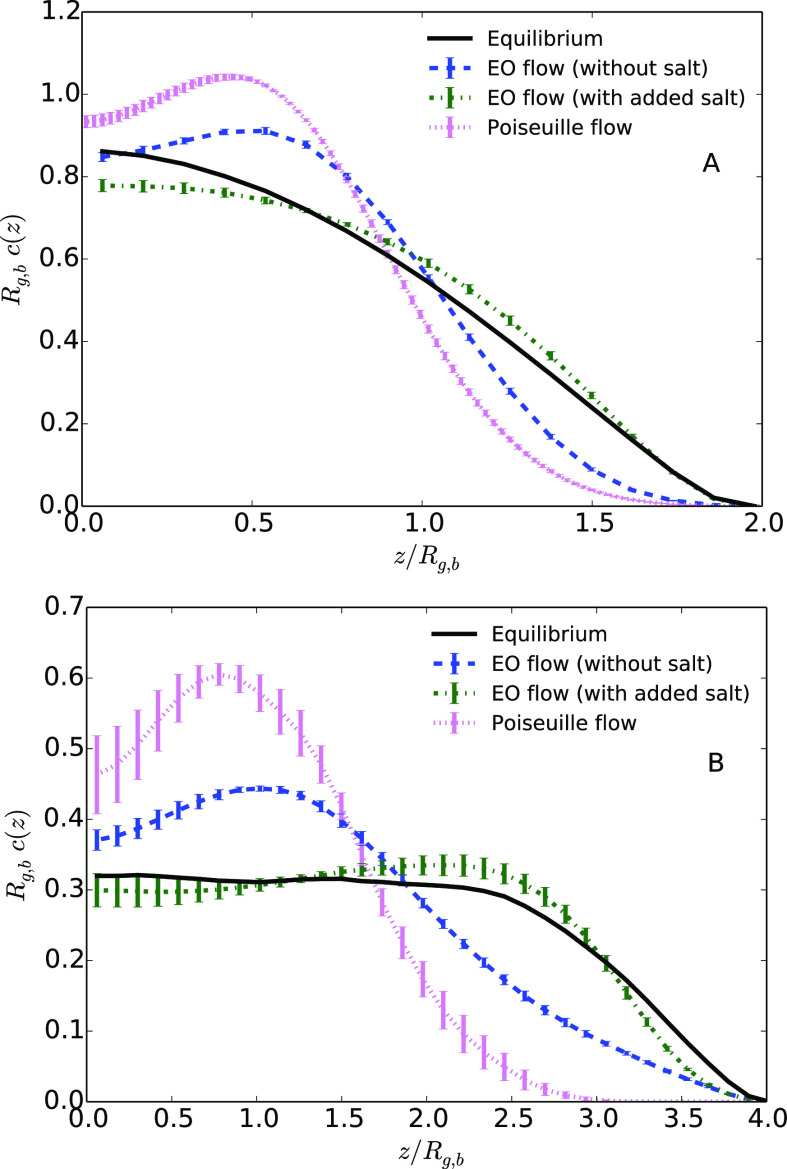
Average monomer density as a function of the distance to the center of the channel *z* divided by the average gyration radius in bulk, *R*_g,b_, for a distance *L*_*z*_ = 4.1*R*_g,b_ between the solid walls (upper graph) and for *L*_*z*_ = 8.2*R*_g,b_ (lower graph) at equilibrium or under flow (cases with the highest maximum velocity).

Figure 3B shows the case of a polymer under an EO flow with added salt, i.e., in a situation where the ionic charge, and thus the shear rate, is more concentrated close to the walls of the channel. In such a case, the flow velocity rapidly increases close to the wall, resulting in an overall flow profile close to a plug flow. The comparison with the results presented in Figure 3A shows a clear influence of salt concentration on the hydrodynamic focusing of the polymer. In the presence of salt, the electroosmotic flow does not affect much the effective interaction with the wall, compared to the equilibrium situation. When the shear is localized at a length scale that is small relative to the size of the polymer (or at least smaller than the polymer), our results suggest that the mechanical stress on the polymer might not lead to significant tumbling nor stretching. As a consequence, the flow enables to push the polymer without affecting its shape as much as a Poiseuille flow, nor restricting the confinement space where it evolves by focusing it far from the surface. We compare in Figure 5 the monomer distributions in every case, under EO and Poiseuille flows, for the highest value of the maximum velocity and for two channel widths. Quantitatively, for the systems under investigation here, the effect of the flow is more pronounced in the case of a Poiseuille flow. In any event, the addition of salt under an EO flow can have a great influence on the position of the polymer compared to the walls. This property of polymer transport under EO flow may have important practical interest.

All previous findings are confirmed by analyzing the asphericity of the polymer in the different cases, as a function of the distance to the center of the channel. The results are shown in Figure 4. The uncertainty of the value becomes high as the monomer density decreases close to the wall. Nevertheless, the trend is clear: first, the polymer is much more aspherical under a Poiseuille flow than under an EO flow, with or without added salt (note that we did not obtain values close to the wall as the polymer is never there); second, there is a small increase of the asphericity close to the surface, compared to the equilibrium case, under an EO flow with added salt, showing that the polymer shape is affected locally by the high shear; and third, there is a small increase of the asphericity in all the channel under an EO flow without salt compared to the case with salt.

### 4.3 Flexible Polymer Interacting with Walls through van der Waals Attractions

**Figure 6:**
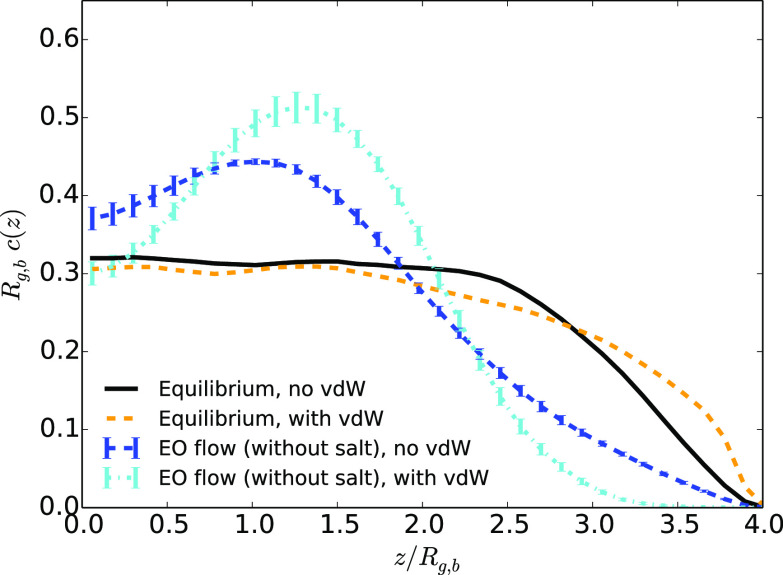
Average monomer density as a function of the distance to the center of the channel *z* divided by the average gyration radius *R*_g,b_ in the case at equilibrium or under an EO flow (with the highest maximum velocity) in the presence or absence of van der Waals attractive interactions between monomers and walls for systems without added salt.

**Figure 7:**
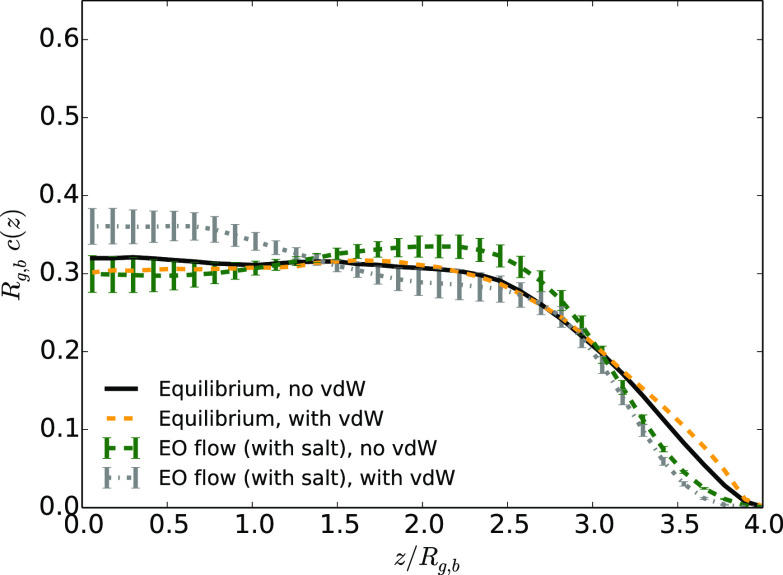
Average monomer density as a function of the distance to the center of the channel *z* divided by the average gyration radius *R*_g,b_ in the case at equilibrium or under EO flow (with the highest maximum velocity) in the presence or absence of van der Waals attractive interactions between monomers and walls for systems with added salt.

**Figure 8:**
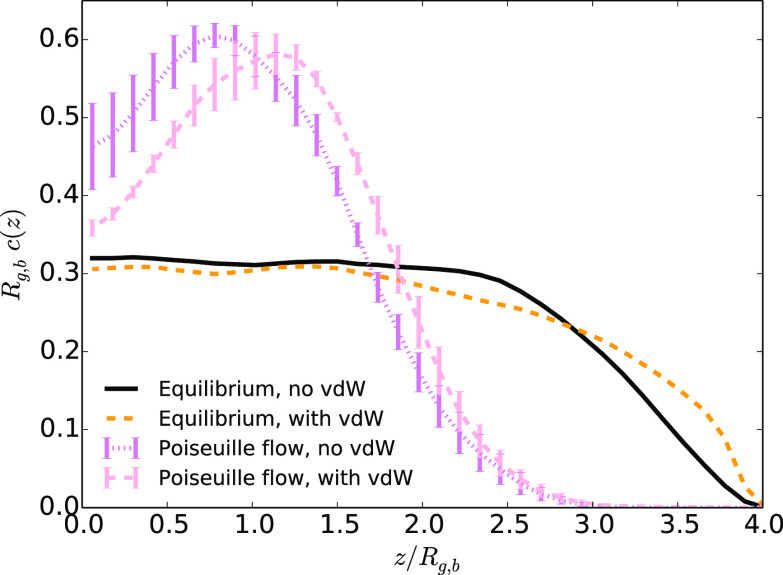
Average monomer density as a function of the distance to the center of the channel *z* divided by the average gyration radius *R*_g,b_ in the case at equilibrium or under Poiseuille flow (with the highest maximum velocity) in the presence or absence of van der Waals attractive interactions between monomers and walls for systems without added salt.

We now turn to simulations for which the hydrodynamic focusing might be balanced by van der Waals attractive interactions with the walls. The results are shown in Figure 6–8. In all cases, the distance between walls is equal to *L*_*z*_ = 8.2 *R*_g,b_. The results obtained at equilibrium, i.e., in the absence of flow, are displayed in each figure to provide a reference. Note that, in Figures 6 and 8, the vdW attraction is in both cases computed with an inverse Debye length κ = 0.23*a*_0_^–1^ (situation without added salt), whereas in Figure 7 it is computed with an inverse Debye length κ = 0.47*a*_0_^–1^ (situation with added salt, *c*_salt_ = 2 mol L^–1^). As a consequence, in the presence of added salt, vdW interactions are more screened than without salt.

**Figure 9:**
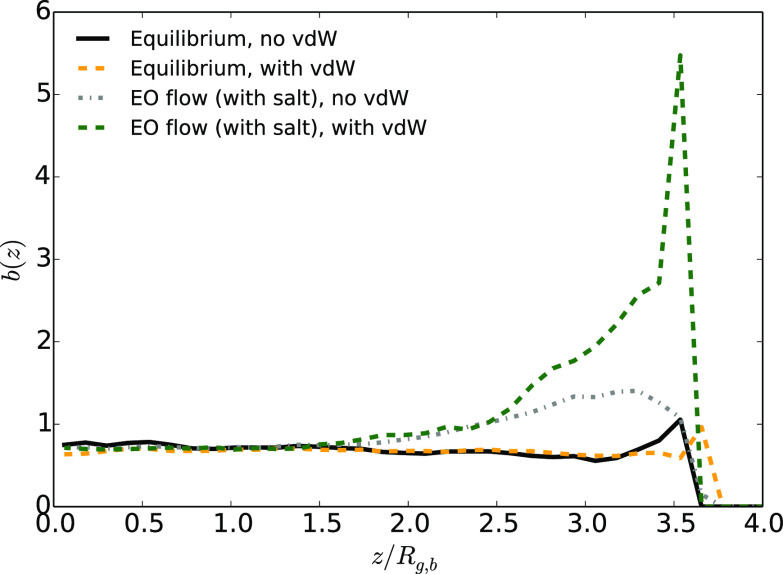
Polymer asphericity as a function of the distance to the center of the channel *z* divided by the average gyration radius in bulk, *R*_g,b_, for a distance of *L*_*z*_ = 8.2*R*_g,b_ between the solid walls at equilibrium and under an EO flow (case with the highest maximum velocity) in the presence or absence of vdW interactions between the monomers and the walls for systems with added salt. The error bars on the results obtained are not shown, as they are high for *r*/*R*_g,b_ > 2.

It appears that at equilibrium, the vdW attraction for the wall does not significantly affect the properties of the polymer: the monomer density (Figures 6–8), the gyration radius, and the polymer asphericity (Figure 9) are the same with attractive or purely repulsive walls. Under electroosmotic flow, we obtain a rather counterintuitive result: When we add an attractive contribution to the interaction between the monomers and the walls, the monomers are actually more repelled from the walls (see Figure 6). Indeed, the monomer density decreases close to the wall (for distances *z*/*R*_g,b_ between 2.3 and 4) when the vdW attraction is added, and its maximum value increases, at *z*/*R*_g,b_ close to 1.3. Under an EO flow without added salt, the monomer density in the presence of attractive vdW interactions is similar to that obtained under a Poiseuille flow without attraction for the wall, displaying a strong deviation from the equilibrium density profile close to the wall. The monomer density in the middle of the pore is, however, lower for the case with vdW interactions than for the case with purely repulsive walls. This might seem counterintuitive, as the range of the vdW interaction is very small compared to the distance to the wall in the middle of the channel. For a better insight, we need to analyze more precisely the shape of the density profile. Why is the maximum of the monomer density shifted from the center of the channel? When the polymer rotates under flow, the alignment of the monomers along the streaming lines concentrates the monomers within cylindrical tubes parallel to the flow. When the polymer crosses the zero shear plane (*z* = 0), the zone of maximum velocity of the solvent, the overall mechanical stress on the polymer decreases as the velocity gradient felt by the chain drops. Then, the chain does not tumble as much (since the shear changes sign) and the monomers are less concentrated. That is why we see a peak of the density close to *z* = *R*_g,b_ in the absence of vdW attractions. Nevertheless, if the polymer is elongated, this peak can be shifted toward larger *z* values. In the case of a polymer under EO flow, with vdW attractions with the walls, the peak is slightly shifted, at *z* = 1.3*R*_g,b_, reflecting an elongation of the polymer in this case, a hypothesis that is confirmed through the computation of the asphericity.

In the presence of an EO flow with added salt, the aforementioned effect occurs close to the wall (see Figure 7). In all cases with added salt, the influence of hydrodynamically induced effective forces is smaller than that in the case without added salt, except close to the wall, where the hydrodynamic focusing is induced by a strong shear. By comparing the plots at equilibrium and under flow, it is striking that lift forces, below a distance of about *R*_g,b_ from the wall, are magnified by the presence of van der Waals attractions for the walls. For the systems under Poiseuille flow, the influence of a polymer–wall attraction is completely different (see Figure 8). There is no additional repulsion/focusing in the presence of vdW interactions in this case.

While van der Waals interactions are usually invoked to explain how molecules may stick together, we have discovered a situation for which their presence finally leads to an effective repulsion. In order to get more insight into this unexpected phenomenon, we computed the asphericity, which is the structural parameter that is the most related to the polymer stretching. The results are shown in Figure 9. The observations related to the monomer density perfectly correlate with asphericity. In the case of EO flow with added salt, when the walls attract the polymer through vdW interactions, the asphericity increases and is close to the values obtained under a Poiseuille flow (see Figure 4). It thus seems that there is a strong synergistic effect between effective hydrodynamic interactions arising from the shear and van der Waals interactions. VdW attractions between monomers and walls alone are not strong enough to affect the shape of the polymer (they do not influence the asphericity at equilibrium). Hydrodynamic shear alone, under an EO flow, slightly affects the asphericity, but much less than under a Poiseuille flow. The effects of conservative vdW interactions and hydrodynamic shear add up non linearly to stretch the polymer. The lift force is then much more pronounced, and it overcomes the attractive effect of vdW forces within the nonequilibrium effective interaction between the polymer and the wall.

The influence of short-range vdW interactions at distances larger than their cutoff distance is a signature of nonequilibrium systems evolving at multiple time scales. If vdW forces and lift forces stretch the polymer and increase its asphericity close to the wall, the structure of the polymer may remain elongated for a long relaxation time (comparable to the Zimm time defined for bulk polymers). Conversely, the tumbling time scale and the time scale at which hydrodynamic shear may focus the polymer away from the wall can be significantly faster. The ratio of these two time scales, the polymer folding/unfolding time scale divided by the hydrodynamic flow time scale, defines the Weissenberg number *Wi*. The polymer time scale can be identified with the slowest mode within the Zimm model of the dynamics of a polymer chain.^13,26^ The time scale τ_Z_ associated with this mode reads:
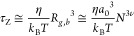
16with *N* being the number of monomers and the Flory exponent of a self-avoiding chain, ν ≅ 0.588. The flow time scale can be expressed as the inverse of the average shear rate, *L*_*z*_/*v*_max_, where *v*_max_ is the maximum value of the solvent velocity and *L*_*z*_ is the distance between the two solid–fluid interfaces. This is an approximation, as the angular velocity may not exactly scale as the shear rate in this regime,^14^ and correction factors may be used. Nevertheless, we do not here intend to look at the quantitative dependence of structural quantities on *Wi*, but rather to explore qualitatively an unexpected phenomenon.

In the systems investigated here, we are in a regime of moderate Weissenberg numbers (*Wi* ≈ 17). In such cases, even if transient interactions between the polymer and the wall surface may slow down the monomers at the interface for a finite period of time, the extension of the chain induced by these interactions may remain for a much longer period of time, as the polymer relaxation time scale is greater than the transport time scale of the polymer. It results in a long-lived increase of the polymer asphericity toward a value that is much greater than the equilibrium asphericity of the chain. In this regime, the repulsive lift forces are considerably magnified by the polymer extension due to vdW forces, leading to a strong hydrodynamic focusing. The polymer is not stuck to the wall, but it keeps a memory of the few attractive interactions even when it has moved far from the wall.

Can Poiseuille flow cause a similar behavior? We did not observe for systems under Poiseuille flow that adding attractive conservative forces may lead to an effective repulsion. Nevertheless, such behavior may occur in regions of parameter space we did not explore. However, we should emphasize that, for a fixed input of energy to make a fluid flow, electroosmosis, by concentrating the hydrodynamic constraints at the interface, may couple short-ranged conservative forces and shear-driven forces more strongly than Poiseuille flow does.

### 4.4 Hydrodynamic Focusing of More Persistent Chains under Flow

We now turn to the case of polymers with additional intrachain interactions (bending potential) that increase their persistence length. For these stiff polymers, the bending potential is chosen so that the persistence length gets a value *L*_*p*_ ≅ 10σ. The gyration radius of these polymers is significantly larger than that of the previously studied polymers (flexible polymer). The bulk gyration radius of stiff polymers is *R*_g,b_ = 10.52*a*_0_, to be compared with the value for a flexible chain of *R*_g,b_ = 4.17*a*_0_.

**Figure 10:**
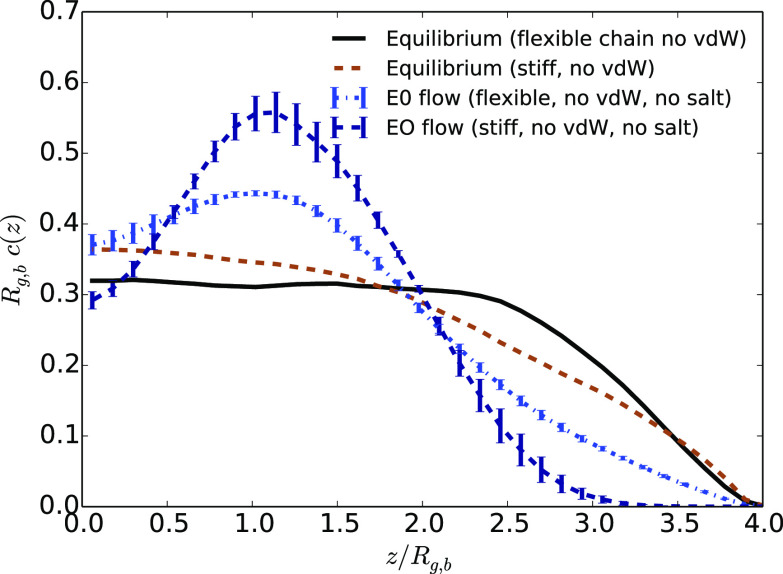
Average monomer density as a function of the distance to the center of the channel *z* divided by the average bulk gyration radius of the flexible chain *R*_g,b_ in the case at equilibrium or under EO flow without added salt (with the highest maximum velocity) for stiff chains and flexible ones without vdW interactions between the monomers and the walls.

We first consider the case without van der Waals attraction between the monomers and the walls. As shown in Figure 10, the equilibrium distribution of monomers in the channel is not qualitatively affected by the stiffness of the chain. At equilibrium, the range of the effective interaction between the monomers and the wall is higher for stiff polymers. This is an expected consequence of the increase of the gyration radius: For a distance between a monomer and the wall that is larger than the gyration radius of the flexible chain and lower than that of the stiff chain, the wall affects more the configurational entropy of the stiff chain than that of the flexible chain.

In the presence of an electroosmotic flow, we find that hydrodynamic focusing is more present for stiff polymers than for fully flexible ones. In particular, close to the wall, the influence of the flow on the effective repulsion with the wall is more pronounced for stiff chains. In contrast, under a Poiseuille flow, stiff chains and flexible ones behave very similarly (not shown) and are both similar to the case of stiff chains under EO flow.

**Figure 11:**
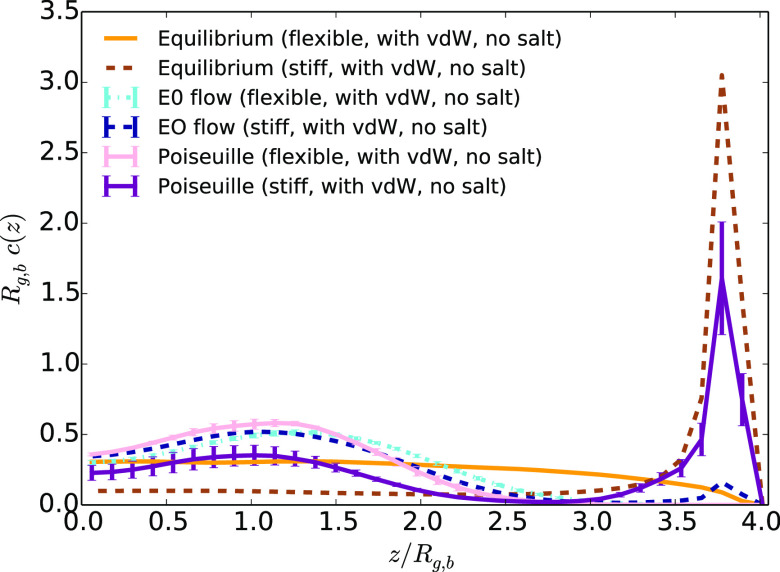
Average monomer density as a function of the distance to the center of the channel *z*,divided by the average bulk gyration radius *R*_g,b_ at equilibrium and under EO or Poiseuille flow (with the highest maximum velocity) for stiff and flexible chains for systems without added salt and with vdW attractions between monomers and the walls.

**Figure 12:**
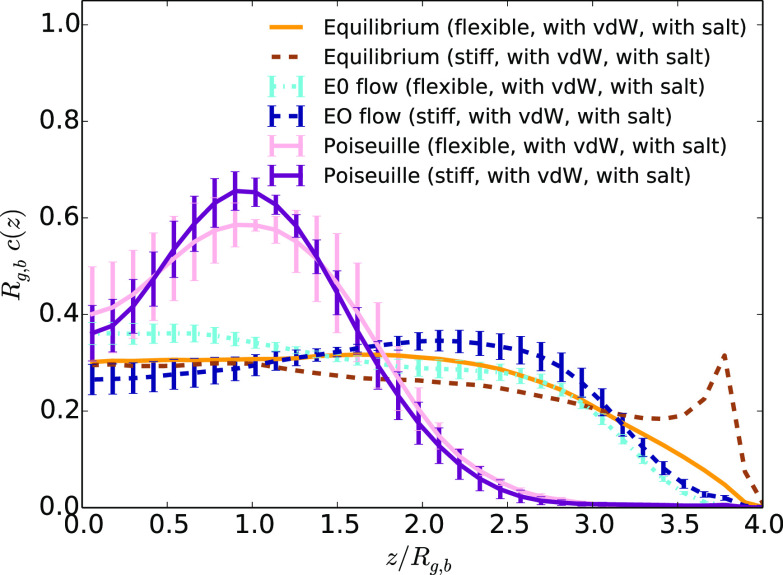
Average monomer density as a function of the distance to the center of the channel *z* divided by the average bulk gyration radius *R*_g,b_ at equilibrium and under EO or Poiseuille flow (with the highest maximum velocity) for stiff and flexible chains for systems with added salt and with vdW interactions between monomers and the walls.

The addition of van der Waals attractions with the walls has a strong influence on the monomer distribution in the case of stiff polymers, as shown in Figure 11 for the case of an EO flow without added salt. In contrast to the case of flexible chains, an attraction *w* ≅ *k*_B_*T* per monomer is sufficient to induce a strong adsorption of the chain on the interface, as revealed by a peak of the monomer density at the wall. In the presence of added salt, vdW interactions are screened. The density peak is still visible, but it is less intense (see Figure 12). Such influence of the stiffness is an expected consequence of the lower entropy of a stiffer chain in bulk. The entropic cost of adsorbing a stiff chain is therefore lower than the one for flexible chains. For stiff chains, this entropic barrier can be overcome by vdW attractions. Under an external field acting on the solvent, the shear induces lift forces that may desorb the polymer. In the case without salt, for which the vdW interactions are weakly screened, the shear induced by the Poiseuille flow at the interface partially desorbs the polymer. In contrast, in the presence of an EO flow due to an external electric field, the monomer density peak almost disappears. The electroosmotic flow may shear the polymer so strongly that the resulting lift forces overcome vdW attractions with the walls, and drives it toward the center of the channel. Finally, in the presence of added salt, the flow poorly focuses the polymer, but the strong shear at the interface enables to release the monomers from adsorption.

### 4.5 Transport Velocity of the Polymer

Finally, we computed the average velocity of the monomers in the direction of the flow. Under a Poiseuille flow, for all systems with flexible polymers, the mean velocity of monomers is very close to the maximum velocity of the fluid (⟨*v*_mono_⟩ ≃ 0.9*v*_max_) and larger than the mean velocity of the fluid (which is equal to 2*v*_max_/3 for a Poiseuille flow), as the polymer is on average close to the center of the channel. The situation is similar for stiff polymers, except when monomers interact with the solid walls through weakly screened van der Waals interactions. In the latter case, the mean velocity of the polymer is smaller than the mean velocity of the fluid (⟨*v*_mono_⟩ ≃ 0.6*v*_max_). These results are consistent with the stronger density of monomers close to the wall when the influence of van der Waals interactions dominate over the hydrodynamic focusing induced by the Poiseuille flow.

Under EO flow, the average velocity of the fluid is larger than with Poiseuille flow (⟨*v*_fluid_⟩ ≃ 3*v*_max_/4 without salt, ⟨*v*_fluid_⟩ ≃ 0.88*v*_max_ with salt). Nevertheless, in the absence of vdW interactions, the mean velocity of the monomers ⟨*v*_mono_⟩ is lower than that in the case of a Poiseuille flow (⟨*v*_mono_⟩ ≃ 0.6*v*_max_ for flexible polymers). This is consistent with our previous observations that the polymer is less focused with EO flow, so that the chain may be more slowed down by the presence of monomers close to the wall. The mean velocity of flexible polymers under EO significantly increases under the influence of attractive interactions (⟨*v*_mono_⟩ ≃ 0.93*v*_max_ without salt and ⟨*v*_mono_⟩ ≃ 0.99*v*_max_ with salt). Again, this is consistent with our finding that vdW attractions increase hydrodynamic focusing under EO flow. This result underlines the striking paradox of these systems: adding attractive interactions with the wall enables the polymer to flow faster. Lastly, for stiff polymers under EO flow, the impact of vdW interactions on the polymer under EO flow is low, which is corroborated by similar values of the mean velocity. In all cases, ⟨*v*_mono_⟩ is larger than 0.9*v*_max_.

## 5 Conclusions

Based on a series of mesoscopic simulations of a single polymer in a slit pore, we have found that Poiseuille flows and electroosmotic flows can lead to quantitatively and qualitatively different behaviors of the chain. In contrast to Poiseuille flow, electroosmosis offers more degrees of freedom, as the shear distribution can be easily tuned by changing the salt concentration. In particular, we have discovered a singular phenomenon in the presence of an electroosmotic flow: Nonequilibrium forces lead to a strong repulsion of the polymers from the solid surfaces, when the monomers are attracted by the surfaces through conservative forces. The polymer is concentrated close to the center of the channel, where the solvent flows faster, thus resulting in a larger streaming velocity. In short, adding glue on the walls makes macromolecules speed up. This discovery opens up many perspectives for the manipulation of polymers under flow, the control of which requires a fine understanding of the mechanisms and an extensive exploration of the phenomenon.

A major objective of the research in nanofluidics is to achieve a better selectivity in manipulating the fluxes of nano-objects and in particular of biopolymers. Many of these polymers can be manipulated, separated, sequenced, or chemically modified within nanoporous environments, i.e., natural or human-made structures comprising nanometer sized pores.^47^ Such processes have benefited from the recent progress in the ability of chemists to build nanopores and nanochannels, mainly inspired by biomaterials. As these research areas and applications mature, there are many fundamental challenges that need to be addressed to unlock the full potential of these nanodevices. Better understanding of the molecular transport at play could inspire new synthetic devices. We have here provided a new perspective on the use of electroosmosis within such devices. In future works, we attend to provide a robust theoretical framework for electroosmotically driven polymer solutions in nanopores.
